# Isobaric Crystallization of Aqueous Magnesium Chloride Solutions at Icy Moon Conditions

**DOI:** 10.1021/acs.jpca.6c05426

**Published:** 2026-10-22

**Authors:** Milz L. Beaumont, Jinjie Li, Miroslava Novoveska, Ines E. Collings, Craig L. Bull, Christoph G. Salzmann

**Affiliations:** † Department of Chemistry, 4919 University College London, 20 Gordon Street, London WC1H 0AJ, United Kingdom; ‡ Natural History Museum, Cromwell Road, South Kensington, London SW7 5BD, United Kingdom; § ISIS Pulsed Neutron and Muon Source, STFC Rutherford Appleton Laboratory, Harwell Campus, Harwell OX11 0QX, United Kingdom

## Abstract

Subsurface oceans inside icy moons are expected to contain dissolved salts whose crystallization controls the mineralogy of their hydrospheres and potentially their surfaces. Here, we use *in situ* neutron diffraction to investigate the isobaric crystallization of a nearly saturated MgCl_2_/D_2_O solution between ambient pressure and 0.4 GPa. At ambient pressure, substantial supercooling leads primarily to MgCl_2_·12D_2_O, whereas increasing the pressure to only 0.1 GPa fundamentally changes the crystallization pathway, yielding the recently discovered MgCl_2_·10D_2_O-II as the dominant hydrate throughout the 0.1–0.4 GPa range. The simple isobaric route enables reliable preparation of decahydrate-II, opening the door to future studies, including spectroscopic characterization relevant to its remote detection on icy moons. We further determine a new thermal equation of state and reveal a curious contrast between its responses to pressure and temperature: the most compressible crystallographic direction exhibits the smallest thermal expansion. These findings demonstrate the importance of pressure for understanding salt-hydrate crystallization and icy-moon mineralogy.

## Graphical abstract

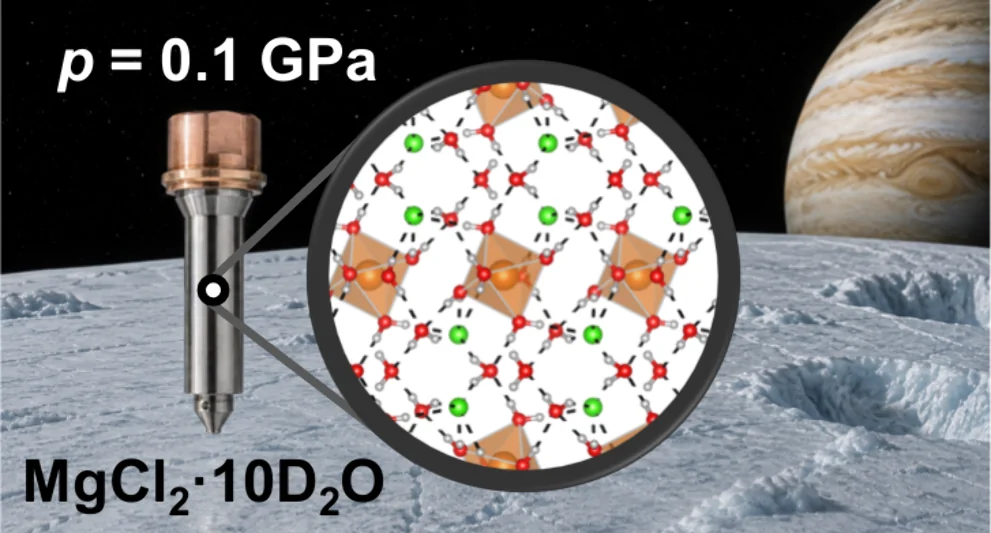

## Introduction

Hidden beneath ice shells up to several hundreds of kilometers thick, vast subsurface oceans are thought to exist on several of Jupiter’s Galilean moons.^1^ Following the pioneering observations by the Galileo and Juno spacecraft, their exploration is now entering a new era: NASA’s Europa Clipper and ESA’s JUICE (JUpiter ICy moons Explorer) are currently en route to Jupiter, where they will investigate Europa, Ganymede, and Callisto in unprecedented detail, while China’s planned Tianwen-4 mission is expected to launch around 2030 to explore the Jovian system, with a particular focus on Callisto. Interpreting the observations from these missions requires a detailed understanding not only of pure water ice, whose physical behavior has been extensively investigated,^2^ but also of the multicomponent salty solutions expected within the subsurface oceans and ice shells. Salts are particularly important because crystallization of ocean-derived brines produces assemblages of ice and salt hydrates whose composition, structure, and stability depend strongly on temperature, pressure, salt concentration, and thermal history. Consequently, the mineralogy of material transported through the ice shells through upwelling processes and ultimately exposed at their surfaces can provide information about both the ocean chemistry and the geological processes connecting the oceans to the surface.

Recent discoveries in the NaCl–H_2_O system illustrate how incomplete our understanding of such processes remains. Two previously unknown hyperhydrated NaCl phases have been identified under conditions relevant to icy moons, including 2NaCl·17H_2_O and NaCl·13H_2_O.^3^ Rapid crystallization can furthermore produce a metastable NaCl hydrateat ambient pressure, distinct from the thermodynamically stable hydrohalite (NaCl·2H_2_O).^4,5^ More recent work has shown that the formation of stable, metastable, and even amorphous NaCl–H_2_O phases depends systematically on both the brine concentration and cooling rate, demonstrating that salt-hydrate mineralogy can preserve a record not only of the ocean composition but also of the thermal and geological history of ocean-derived material.^5^

Magnesium chloride (MgCl_2_), which is the second most abundant salt in Earth’s oceans, is also of interest in the context of the icy moons.^6^ Geochemical models and aqueous differentiation experiments using carbonaceous-chondrite precursors predict that subsurface oceans may contain substantial concentrations of Mg^2+^ and Cl^–^, alongside Na^+^, Ca^2+^, K^+^, SO_4_^2–^, and CO_3_^2–^ species.^7–10^ Observational evidence also points toward chloride-rich surface materials. In particular, ground-based near-infrared observations of fresh terrains on Europa and Ganymede have been interpreted as evidence for chloride salts and magnesium-containing chlorinated hydrates, including MgCl_2_·2H_2_O.^11–13^ Laboratory experiments on frozen brines containing Na^+^, Mg^2+^, Cl^–^, and SO_4_^2–^ further demonstrate that MgCl_2_ hydrates can crystallize under relevant cryogenic conditions, with MgCl_2_·12H_2_O identified among the expected salt-hydrate mineral assemblages.^14,15^ In addition, spectroscopic measurements of MgCl_2_·*n*H_2_O with *n* = 1, 2, 4, 6, 8, and 12 have established systematic changes in the near-infrared spectra with the hydration state, providing reference data relevant to the interpretation of remote-sensing observations of Europa.^16^ Collectively, these geochemical, observational, and laboratory results provide strong motivation for establishing the structures, stabilities, and spectroscopic properties of MgCl_2_ hydrates under the low-temperature and high-pressure conditions encountered within icy moons.

Previous high-pressure studies have shown that the MgCl_2_–H_2_O system displays considerable structural diversity under pressure, including the formation of MgCl_2_·10H_2_O-I, MgCl_2_·7H_2_O, and a high-pressure polymorph of MgCl_2_·6H_2_O.^17–19^ More recently, Collings et al. identified a second decahydrate polymorph, MgCl_2_·10H_2_O-II, which is stable over a broad range of low-temperature and high-pressure conditions relevant to icy-moon interiors, extending up to 2.11(3) GPa at 220(2) K.^20^ The phase was observed upon decompression down to 0.03(14) GPa at 234(11) K, suggesting that it may persist during cold decompression and potentially be transported to the surfaces of icy moons.^20^ These observations are particularly relevant to large icy moons, where pressures within the hydrosphere can extend into the gigapascal range.^1^ Consequently, the salt-hydrate assemblage present at depth may differ substantially from that predicted solely from ambient-pressure phase behavior, and the detection of high-pressure MgCl_2_ hydrates at the surface could provide evidence for endogenous material and vertical transport from deeper regions of the hydrosphere.^20^

Overall, magnesium chloride exhibits a rich variety of hydrate phases, MgCl_2_·*n*H_2_O, with hydration states that depend strongly on temperature and pressure.^21^ At ambient pressure, the known crystalline hydrates have *n* = 1, 2, 4, 6, 8, and 12, as shown in the binary MgCl_2_–H_2_O phase diagram in Figure 1a.^22–27^ The hexahydrate, MgCl_2_·6H_2_O (bischofite), was the first of these hydrates to be structurally characterized, with its crystal structure reported in 1934.^24,25^ Additional hydrate phases become accessible at elevated pressures. These include the original decahydrate MgCl_2_·10H_2_O-I,^17^ a second high-pressure polymorph of the hexahydrate, MgCl_2_·6H_2_O-II,^19^ and, most recently, the second decahydrate polymorph, MgCl_2_·10H_2_O-II.^20^ Thus, the MgCl_2_–H_2_O system is currently known to contain six hydrate stoichiometries at ambient pressure, together with additional high-pressure phases that considerably expand its structural diversity. The crystal structure of the newly discovered MgCl_2_·10H_2_O-II is shown in Figure 1b.^20^

**Figure 1:**
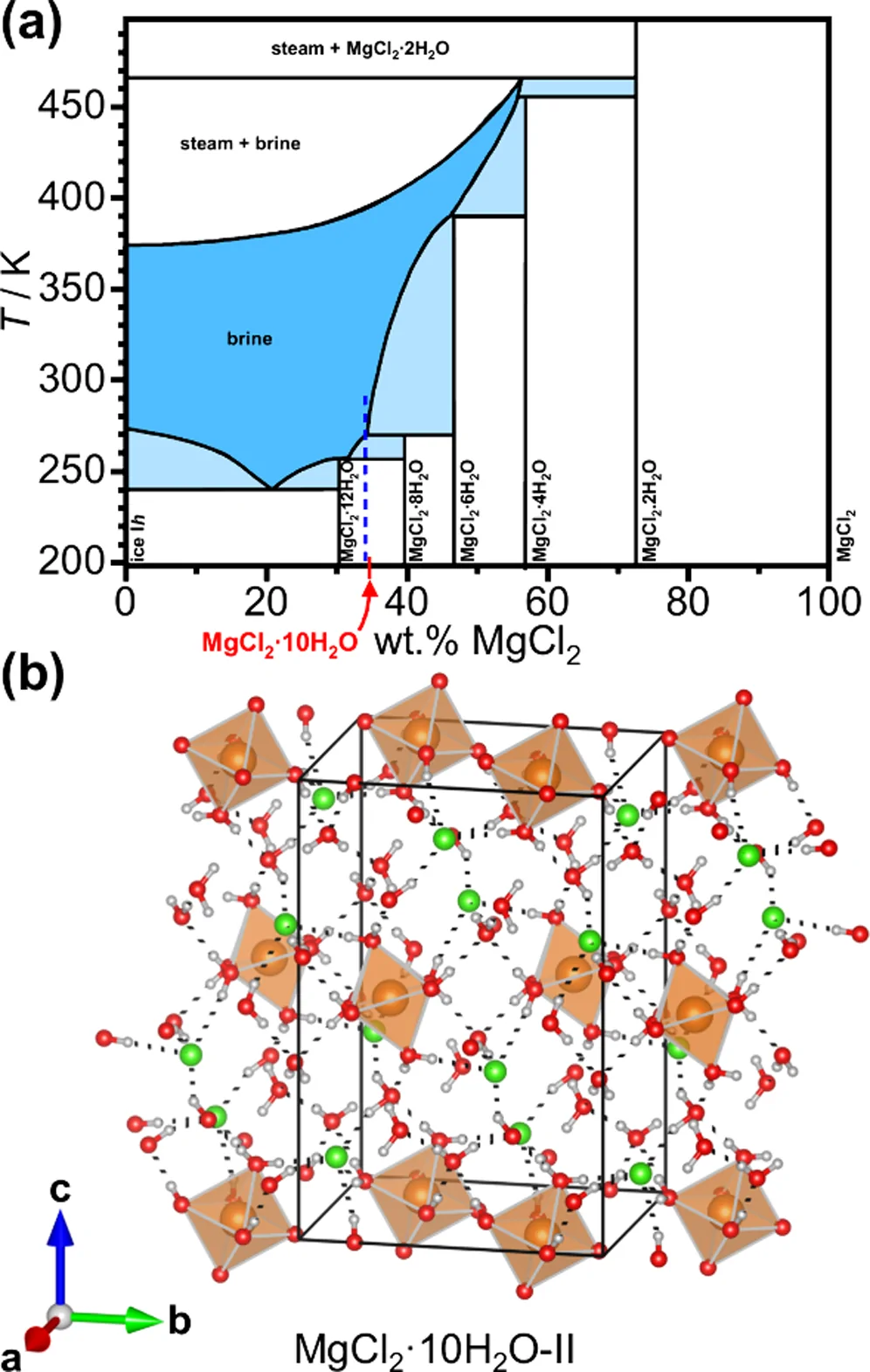
Binary phase diagram of H_2_O and MgCl_2_ at ambient pressure and crystal structure of MgCl_2_·10H_2_O-II.^20,28–31^ (a) The stability region of the unsaturated liquid phase is highlighted in a darker blue, whereas the saturated liquid coexists with solid phases in the light-blue regions. The blue vertical dashed line indicates the concentration of the sample during the neutron diffraction experiment, which is just below the weight percentage of a 1:10 MgCl_2_-to-water mixture (red arrow). (b) Crystal structure of MgCl_2_·10H_2_O-II,^20^ with the unit cell outlined as solid black lines. Oxygen, hydrogen, magnesium, and chlorine atoms are shown as red, white, orange, and green spheres. The covalent O–H bonds are indicated by solid lines, the hydrogen bonding between water molecules and Cl^–^ by dashed lines, and Mg­(H_2_O)_6_ coordination as orange octahedra.

In this work, we investigate the crystallization behavior of a nearly saturated MgCl_2_ aqueous solution under pressure using *in situ* neutron powder diffraction. By cooling the liquid phase under strictly isobaric conditions between ambient pressure and 0.4 GPa, we aim to establish which MgCl_2_ hydrate phases crystallize under pressure–temperature conditions relevant to the hydrospheres of icy moons and to determine how relatively modest increases in pressure affect the crystallization pathways. The strictly isobaric conditions are created with a gas-pressure setup using argon gas as the pressure medium, which represents a major methodological advance compared to previous diamond-anvil-cell experiments where *p*–*T* pathways are difficult to reproduce. Particular attention is given to the recently discovered MgCl_2_·10H_2_O-II polymorph, for which we seek to establish a straightforward isobaric crystallization route and constrain its pressure range of formation toward lower pressures. Furthermore, by determining the unit-cell parameters of MgCl_2_·10D_2_O-II as functions of pressure and temperature, we characterize its compression and thermal-expansion behavior and derive equations of state. Together, these measurements provide new constraints on the formation and physical properties of highly hydrated MgCl_2_ phases that may occur within, and potentially be transported through, the hydrospheres of icy moons.

## Experimental Methods

### Materials

A magnesium chloride (MgCl_2_) solution in D_2_O was prepared by dissolving 19.96 g of anhydrous MgCl_2_ in 43.08 g of deuterated water, yielding a 31.6 wt % solution with a MgCl_2_ mole fraction of 0.0888. The molar MgCl_2_/D_2_O ratio of this nearly saturated solution is 1:10.3.

### Neutron Diffraction Experiment

A cylindrical TiZr cell fitted with a Bridgman seal at the bottom was filled with approximately 2 mL of the MgCl_2_ solution using a plastic pipet. A small amount of ground glass wool was added to promote nucleation, followed by a 0.1 mL layer of silicone oil to act as a barrier and reduce the level of clathrate hydrate formation. Both the ground glass wool and the oil layer are expected to be outside the neutron beam and therefore not to contribute to the diffraction data. The top of the cell was connected to a stainless steel capillary leading to the pressure-generation manifold, with argon serving as the pressure-transmitting medium. Pressures of up to 0.2 GPa were generated by using a diaphragm compressor, while higher pressures were achieved by manually reducing the volume of the pressure medium. To ensure a reliable seal, an initial pressure of 0.1 GPa was applied. The pressure was subsequently maintained within ±0.001 GPa throughout the experiment. The pressure was measured with a Barnbrook HP28 high-pressure transducer located close to the sample cell. The transducer was calibrated by using a UKAS-certified Wika Calibration Rig.

Two thermocouples were attached to the TiZr cell, one above and one below the sample, which was considered thermally equilibrated when the temperature difference between the two sensors was less than 0.05 K. The cell was mounted on a cryostat stick and inserted into a helium cryostat maintained at 290 K on the PEARL beamline at the ISIS Neutron and Muon source.^32^ First, the sample was cooled from 290 to 200 K at 0.1 GPa in temperature steps of typically 10 K. This cooling protocol was subsequently repeated at pressures of 0.2, 0.3, and 0.4 GPa. The pressure was then released to ambient conditions, which was followed by an additional cooling cycle from 290 to 200 K and subsequent reheating. The temperature and pressure errors were estimated as ±0.02 K and ±0.001 GPa.

Neutron powder diffraction patterns were collected at a scattering angle of 90° and typically for 150 μA h of integrated proton current for one measurement. To confirm pressure and temperature equilibration, we confirmed that the Bragg peak positions remained unchanged between the first and second halves of each typically 1 h measurement at a given *p*–*T* point. After the experiments, the TiZr cell was emptied and cleaned and the diffraction data from the empty cell were collected. The empty-can data were smoothed, as shown in Figure S1, and subtracted as a background from all previously recorded data. The background-corrected diffraction data were analyzed using the GSAS software package with the EXPGUI graphical user interface.^33,34^ Unit-cell parameters were obtained using the Le Bail method^35^ and equation-of-state data were determined using the EoSFit7 software.^36,37^

## Results and Discussion

### Ambient-Pressure Crystallization and Melting

The neutron diffraction patterns obtained during cooling and heating of the MgCl_2_ solution at ambient pressure are shown in Figure 2a,b and discussed first because they should be straightforward to interpret in the context of the known ambient-pressure binary phase diagram. The 1:10.3 MgCl_2_/D_2_O isopleth, reflecting the bulk composition of our sample, is indicated by the blue dashed vertical line in Figure 1a. The Bragg peak at approximately 2.9 Å in Figure 2a, which is already present at 290 K, arises from a small amount of cubic structure II Ar clathrate hydrate,^38,39^ formed as a result of using argon gas as the pressure-transmitting medium. These data were collected at the end of the experiment and after decompression from 0.4 GPa.

**Figure 2:**
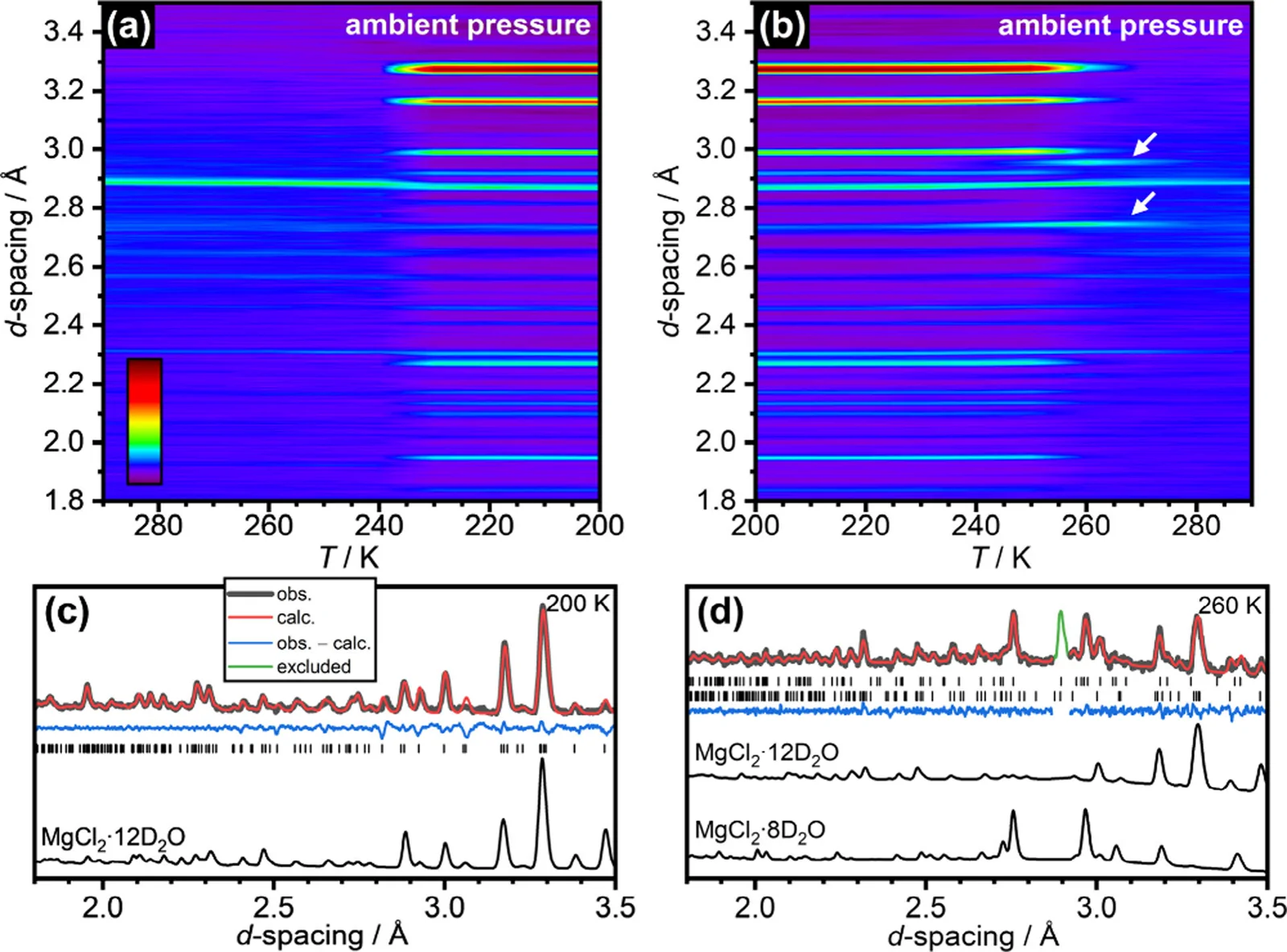
Crystallization and melting of a 1:10.3 MgCl_2_/D_2_O solution at ambient pressure. Neutron diffraction data collected upon (a) cooling from 290 to 200 K and (b) subsequent heating from 200 to 290 K. The arrows in panel b indicate Bragg peaks characteristic for MgCl_2_·8D_2_O. Le Bail fits (red lines) to the neutron diffraction data (black lines) using the unit-cell parameters and space groups of MgCl_2_·12D_2_O and MgCl_2_·8D_2_O after (c) cooling to 200 K and (d) reheating to 260 K.^26,27^ Vertical tickmarks indicate the expected positions of Bragg peaks and the blue lines the differences between the Le Bail fits and the experimental data. The green line shows excluded data due to the Ar clathrate hydrate. Structure-based calculated neutron powder diffraction patterns of the pure phases are shown for comparison at the bottom of panels c and d.

According to the ambient pressure phase diagram shown in Figure 1a, the solution would be expected to crystallize incongruently between 270 and 257 K, yielding increasing amounts of the octahydrate in this temperature range. Below 270 K, the now water-richer liquid phase should then crystallize abruptly to give solid dodecahydrate coexisting with the already formed octahydrate. In our case, substantial supercooling is observed with respect to these processes, and crystallization is only observed between 240 and 230 K, as indicated by the emergence of Bragg peaks. Despite the addition of ground glass wool to the TiZr can, the nucleation of both the dodeca- and octahydrates appears to be a difficult process.

Within the approximately 2 mL sample volume, the hydrate crystals are expected to grow, with preferred orientations reflecting their growth directions, rather than forming a powder with randomly oriented crystallites. As a result, the measured diffraction intensities are affected by the preferred orientation and cannot be fitted using a conventional Rietveld powder diffraction analysis. Because of this, the diffraction data at 200 K in Figure 2c were analyzed using a Le Bail fit, in which the peak positions are determined by the unit-cell parameters and the systematic absences by the space group, while the individual reflection intensities are extracted from the experimental data.^35^ The excellent Le Bail fit based on MgCl_2_·12D_2_O (red line) confirms that the observed diffraction peaks arise from crystallization of the dodecahydrate. For comparison, the structure-based calculated neutron powder diffraction pattern of MgCl_2_·12D_2_O, in which the relative Bragg-peak intensities are determined by the actual crystal structure, is shown as a black line at the bottom of Figure 2c. The peak-shape parameters used in this calculation were those obtained from the Le Bail fit. The differences between the calculated and observed peak intensities indicate a level of preferred orientation, with some of the intensities still reproduced reasonably well.

The water content of the dodecahydrate differs from the initial MgCl_2_/H_2_O ratio of 1:10.3. This means that, in addition to crystalline MgCl_2_·12D_2_O, the sample should contain a salt-rich fraction that did not crystallize and was potentially present as a metastable glassy solution at 200 K. Because such a glassy material would not produce Bragg diffraction, its presence cannot be easily inferred from the diffraction data.

Figure 3a shows the intensities of the strongest Bragg peak of MgCl_2_·12D_2_O at ∼3.27 Å, normalized to the maximum intensity over the 290–200 K temperature range. Again, it can be seen that crystallization takes place between 240 and 230 K upon cooling. Upon heating from 200 to 290 K, the dodecahydrate persists up to 250 K. Heating to 260 K leads to a ∼80% loss of the initial amount of MgCl_2_·12D_2_O judging from the decrease of its peak intensity in Figure 3a. This finding is consistent with the expected peritectic decomposition of MgCl_2_·12D_2_O at 257 K. Heating to 270 K then leads to complete loss of the dodecahydrate.

**Figure 3:**
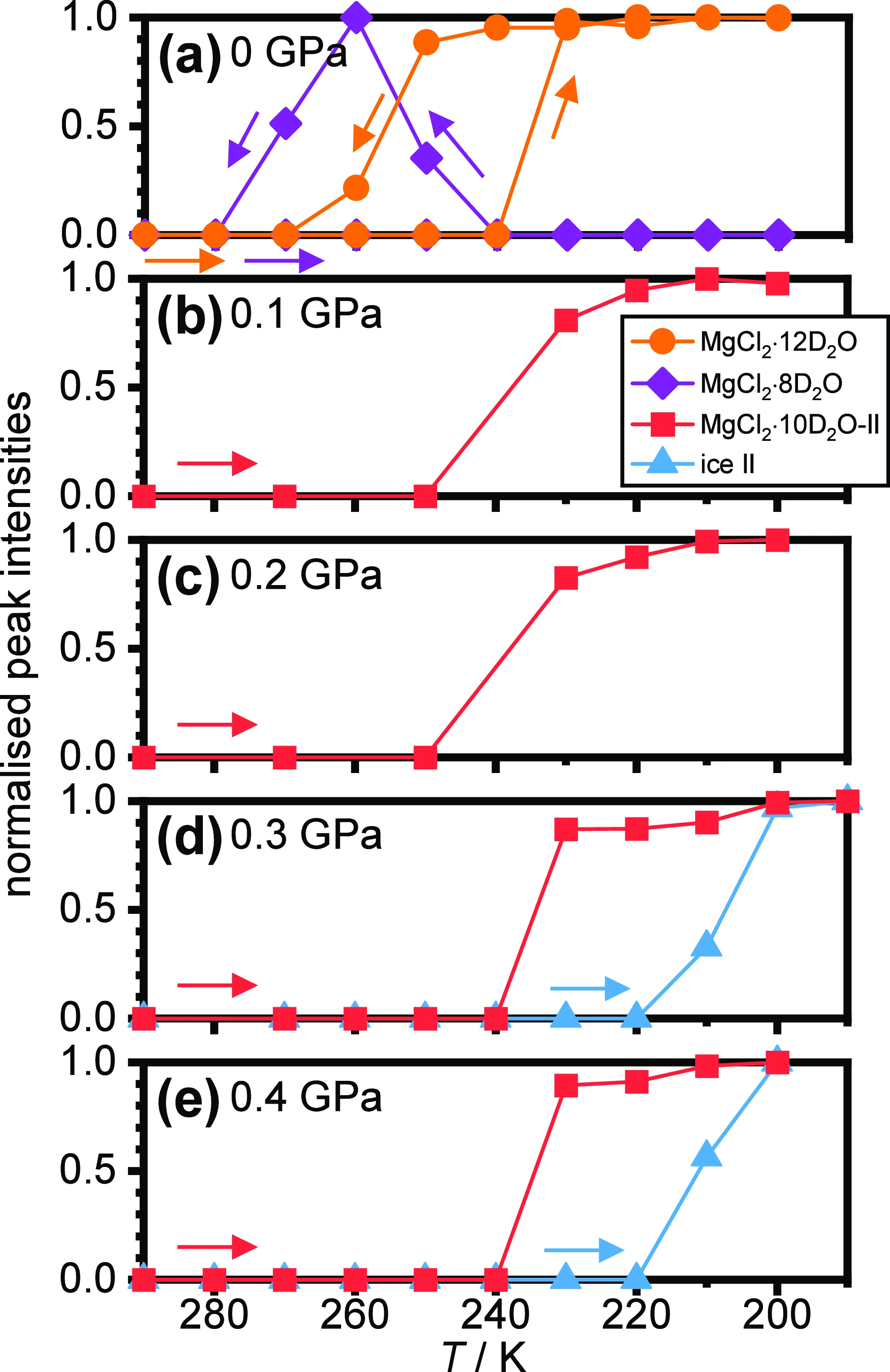
Peak intensities of the most intense Bragg peaks of the various crystalline phases plotted against temperature at pressures of (a) 0, (b) 0.1, (c) 0.2, (d) 0.3, and (e) 0.4 GPa. The peak intensities were normalized with respect to the most intense Bragg peak of a phase within a given temperature range. The crystalline phases include MgCl_2_·12D_2_O (orange circles), MgCl_2_·8D_2_O (purple diamonds), MgCl_2_·10D_2_O-II (red squares), and ice II (blue triangles). Arrows indicate the directions of cooling and heating.

Interestingly, heating leads to the formation of MgCl_2_·8D_2_O, as indicated by the arrows in Figure 2b. The first octahydrate is observed at 250 K, which is below the temperature of the peritectic decomposition of the dodecahydrate, as can be seen in Figure 3. The most intense Bragg peak of the octahydrate increases in intensity while the dodecahydrate has not yet started to undergo peritectic decomposition. This suggests that the octahydrate forms initially from the salt-rich glassy solution mentioned earlier. Upon heating to 260 K, because the amount of the dodecahydrate now decreases, the increase of the intensity of the octahydrate Bragg peak must now be due to the peritectic decomposition of the dodecahydrate. Upon further heating to 270 K, the amount of octahydrate decreases, which is expected in the two-phase region of the phase diagram where the saturated liquid phase and octahydrate coexist. Ultimately, heating to 280 K leads to the disappearance of the octahydrate as the liquidus line is crossed and the remaining octahydrate becomes fully soluble in the liquid phase.

### Isobaric Crystallization in the 0.1–0.4 GPa Pressure Range

Interestingly, a relatively minor increase in pressure from ambient pressure to 0.1 GPa drastically changes the crystallization behavior of the MgCl_2_ solution. The neutron diffraction data collected upon cooling the solution from 290 to 200 K at 0.1 GPa are shown in Figure 4a. A completely new set of Bragg peaks emerges at 230 K, which persists down to 200 K, and these peaks are also observed upon cooling at 0.2, 0.3, and 0.4 GPa, as can be seen in Figure 4b–d. The crystallization temperature does not display a significant pressure dependence as crystallization is observed at around 230 K for all pressures (Figure 3). The slightly higher apparent crystallization onset temperatures at 0.1 and 0.2 GPa in Figure 4a,b are most likely an interpolation artifact caused by the missing data points at 230 K at those pressures, as can be seen in Figure 3b,c.

**Figure 4:**
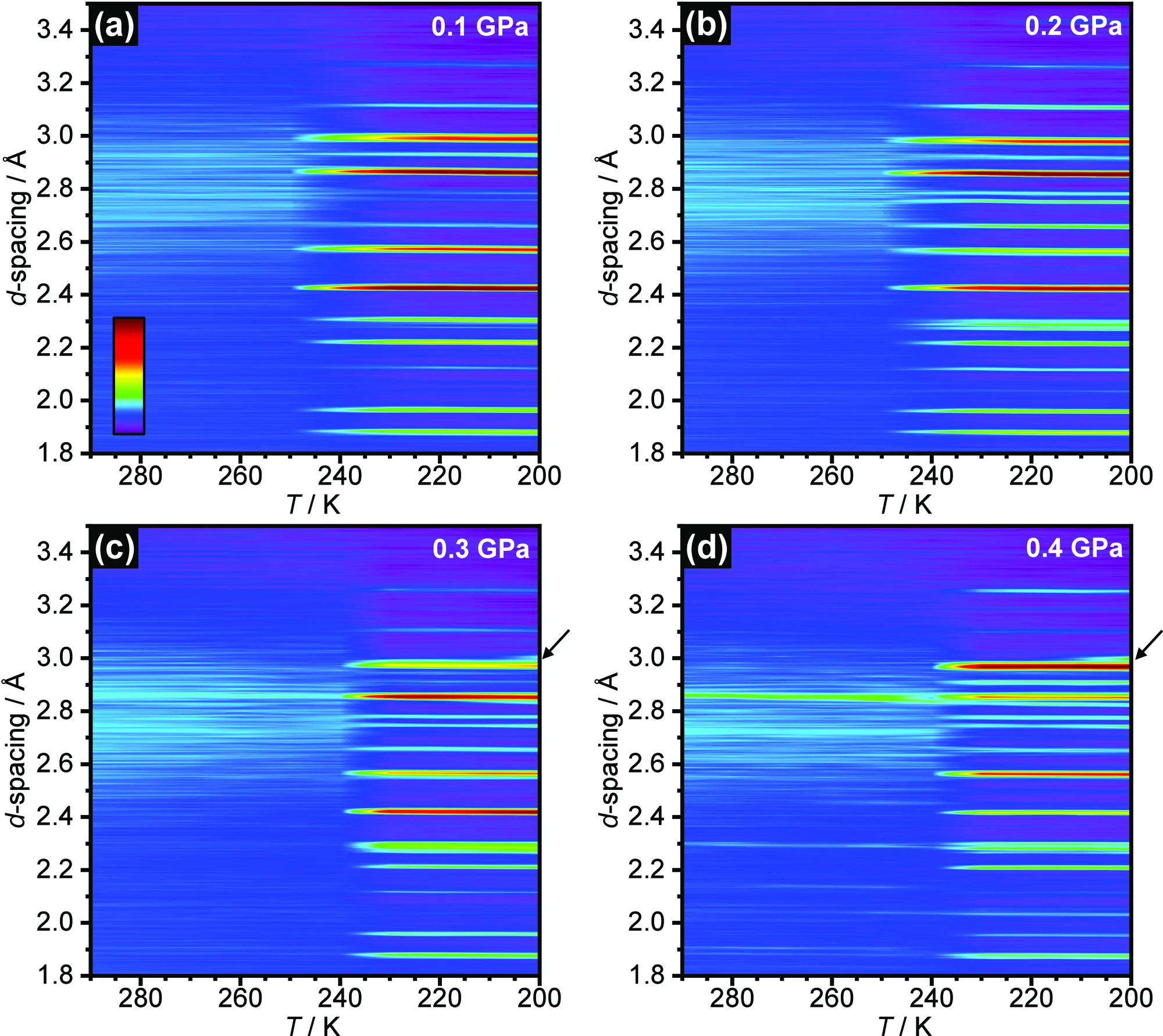
Isobaric crystallization of a 1:10.3 MgCl_2_/D_2_O solution in the 0.1–0.4 GPa pressure range. Neutron diffraction data were collected upon cooling from 290 to 200 K at (a) 0.1, (b) 0.2, (c) 0.3, and (d) 0.4 GPa. Arrows in panels c and d indicate the emergence of a shoulder at one of the main Bragg peaks at low temperatures.

To identify the origin of the newly observed Bragg peaks, diffraction patterns were simulated for the known magnesium chloride hydrate structures, with hydrogen atoms replaced by deuterium where necessary.^17,19–27^ The best agreement with the new reflections was obtained for the recently discovered MgCl_2_·10H_2_O-II phase.^20^ Figure 5 compares the experimental diffraction patterns collected at 200 K over the pressure range 0.1–0.4 GPa with the corresponding calculated pattern of deuterated MgCl_2_·10H_2_O-II. The relative intensities of the experimental reflections vary with the crystallization pressure, indicating a degree of preferred orientation depending on the exact crystallization conditions. This also accounts for the differences in the relative peak intensities between the experimental and calculated diffraction patterns. Nevertheless, the Le Bail fits show that the observed Bragg reflections can be reproduced well using the unit cell and space group of MgCl_2_·10D_2_O-II, supporting the assignment of the phase crystallizing in the 0.1–0.4 GPa range under isobaric conditions to the deuterated decahydrate-II.

**Figure 5:**
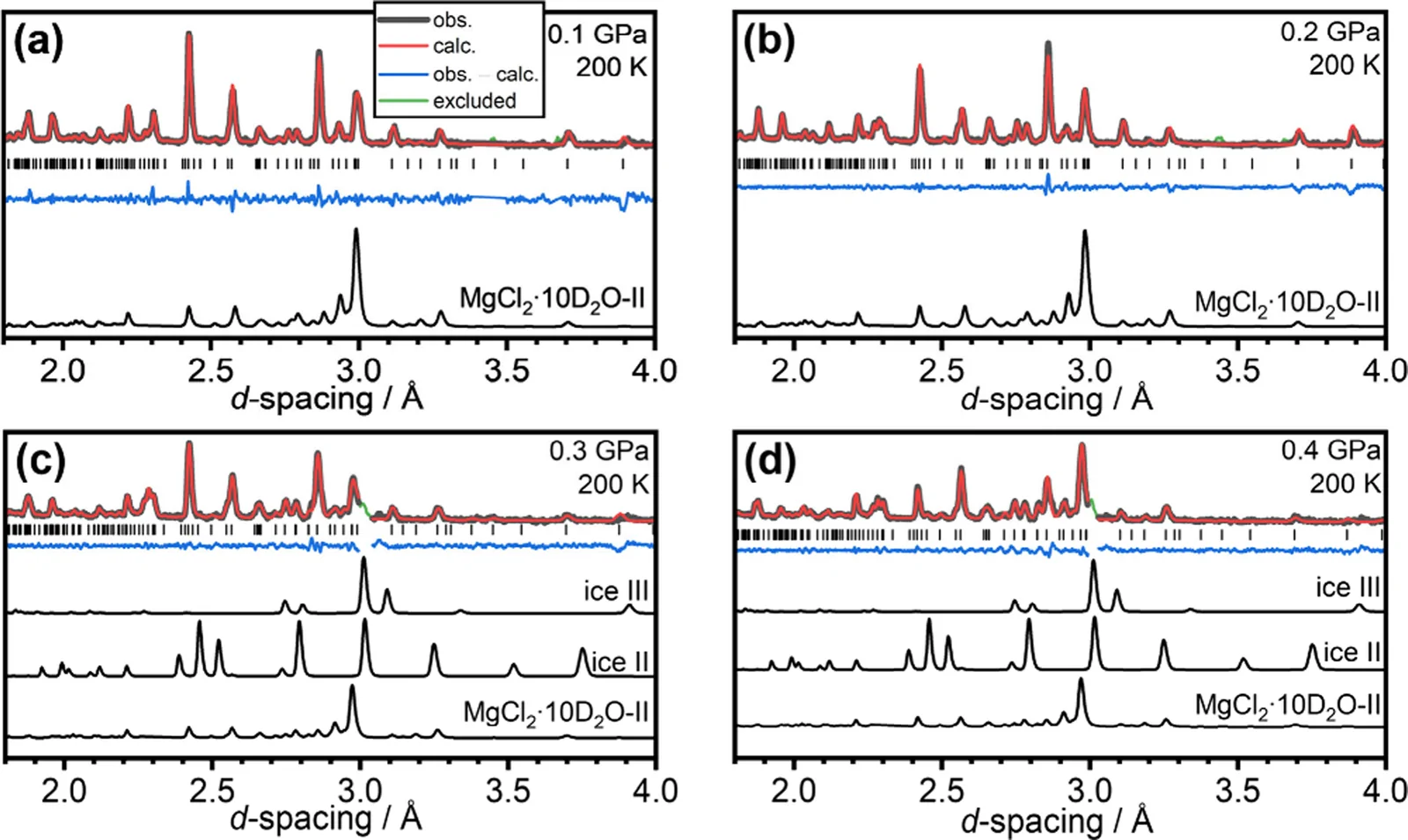
Neutron diffraction data of a crystallized 1:10.3 MgCl_2_/D_2_O solution at 200 K and pressures of (a) 0.1, (b) 0.2, (c) 0.3, and (d) 0.4 GPa. Le Bail fits (red lines) to the neutron diffraction data (black lines) using the structural model of MgCl_2_·10H_2_O-II.^20^ Vertical tickmarks indicate the expected positions of Bragg peaks and the blue lines the differences between the Le Bail fits and the experimental data. Excluded regions in the experimental data are shown with green lines. Structure-based calculated neutron powder diffraction patterns of MgCl_2_·10H_2_O-II,^20^ ice III, and ice II^39^ are shown for comparison at the bottom of panels c and d.

The new MgCl_2_·10H_2_O-II polymorph was recently crystallized independently in two experiments using a diamond anvil cell.^20^ In the first, the sample was initially cooled at 0.16(3) GPa to 149(11) K to form ice I*h*, subsequently compressed to 0.56(14) GPa, and then heated while being decompressed, with MgCl_2_·10H_2_O-II forming at 0.18(14) GPa and 234(11) K. In the second experiment, cooling at 0.30(3) GPa to 211(2) K did not cause immediate crystallization; the sample was therefore compressed to 1.13(3) GPa at 194(2) K and subsequently decompressed while being heated, resulting in the formation of MgCl_2_·10H_2_O-II alongside ice II at approximately 0.56(3) GPa and 220(2) K. These conditions represent crystallization points reached along specific pressure–temperature pathways rather than equilibrium phase-boundary conditions.

In the present study, we demonstrate that the MgCl_2_·10D_2_O-II phase can be crystallized upon cooling the liquid phase under strictly isobaric conditions and at lower pressures than previously achieved.^20^ This contrasts with the previous diamond-anvil-cell experiments,^20^ which followed more complex *p*–*T* pathways and involved partially crystallized samples or individual crystals surrounded by liquid. In our experiments, by contrast, the entire sample was crystallized at constant pressure in the 0.1–0.4 GPa range, demonstrating that MgCl_2_·10H_2_O-II is the dominant MgCl_2_ hydrate formed in this lower-pressure range. Our straightforward isobaric cooling protocol also now provides a reliable preparation route that does not require the complex *p*–*T* histories employed previously.

A minor impurity phase in our samples was indicated by a shoulder or weak diffraction peak on the higher *d*-spacing side of the approximately 2.97 Å reflection of MgCl_2_·10D_2_O-II. These features were observed only in samples crystallized at 0.3 and 0.4 GPa. In Figure 4c,d, their positions are highlighted by arrows. Their intensities, normalized to the maximum diffraction intensity and plotted against temperature, are shown in Figure 3d,e. Together with the diffraction patterns in Figure 4c,d, these data show that the additional feature appears upon cooling at around 210 K. For the sample crystallized at 0.3 GPa, cooling from 200 to 190 K did not result in any additional increase in its intensity. The simulated diffraction patterns of ice II and ice III shown in Figure 5c,d indicate that the strongest reflections of both ice phases are consistent with the position of the observed feature, although the limited diffraction evidence does not permit an unambiguous assignment to either ice II or ice III. Notably, Collings et al. previously observed ice II as a byproduct during the formation of MgCl_2_·10H_2_O-II at 0.56 GPa.^20^ Given the MgCl_2_/D_2_O molar ratio of 1:10.3 in our starting solution, crystallization of the decahydrate-II at higher temperatures is expected to leave behind a small amount of a water-rich residual solution, which subsequently crystallizes as ice II or ice III upon further cooling. These observations would be consistent with the presence of a low-temperature eutectic between MgCl_2_·10D_2_O-II and either ice II or ice III, analogous to the eutectic formed by MgCl_2_·12H_2_O and ice I*h* at ambient pressure (Figure 1a).

Figure S4 shows somewhat lower-quality neutron diffraction data collected upon isobaric heating of MgCl_2_·10D_2_O-II from 200 to 290 K at 0.2 GPa. In contrast to the transient formation of the octahydrate during the ambient-pressure heating experiment (Figure 2b), no additional crystalline phases could be detected before the phase transition to the liquid phase. This observation, together with the reliable crystallization of MgCl_2_·10D_2_O-II across the 0.1–0.4 GPa pressure range, highlights the predominance of this hydrate under the investigated conditions. However, fully confirming its thermodynamic stability may require more extensive thermal annealing at temperatures just below the liquidus line.

### Pressure and Temperature Dependence of the Unit-Cell Parameters and Equation-of-State Analyses

Le Bail fits were carried out on the entire data set of MgCl_2_·10D_2_O-II diffraction patterns to determine the unit-cell parameters (*a*, *b*, *c*, and β) and volumes (*V*). Their pressure dependence over the range 0.1–0.4 GPa at 200, 210, 220, and 230 K is shown in Figure 6. Over the investigated pressure range from 0.1 to 0.4 GPa, all unit-cell parameters vary continuously with pressure, with no discontinuities indicative of a structural phase transition. The unit-cell volume decreases systematically with increasing pressure at all four temperatures, consistent with the persistence of the MgCl_2_·10D_2_O-II structure throughout the investigated *p*–*T* range.

**Figure 6:**
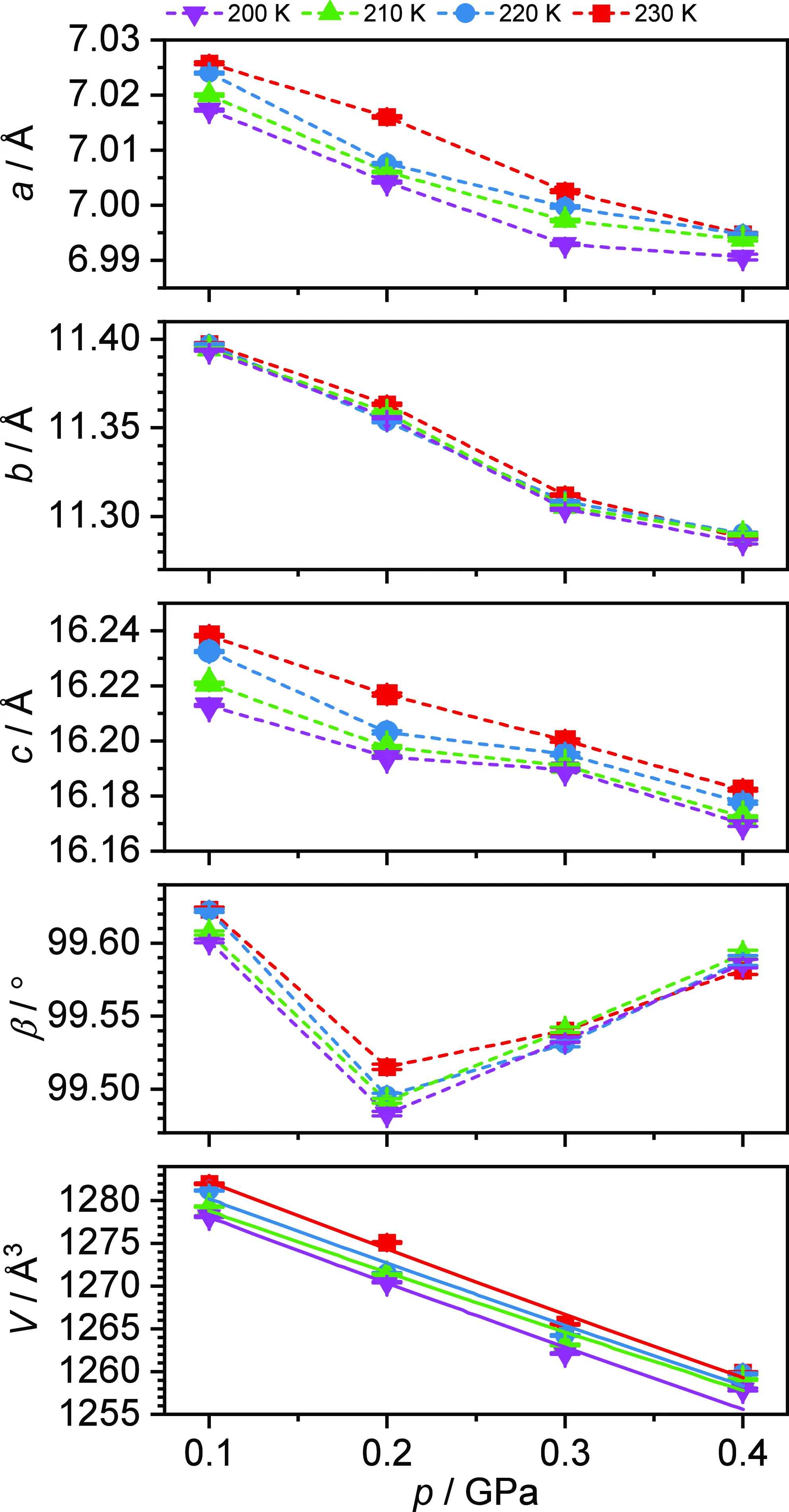
Unit-cell parameters and volumes of MgCl_2_·10D_2_O-II as a function of pressure at temperatures of 200 K (purple downward-pointing triangles), 210 K (green upward-pointing triangles), 220 K (blue circles) and 230 K (red squares). Estimated standard deviations are indicated by error bars and are typically smaller than the symbol sizes. The dashed lines are guides to the eye, whereas the solid lines in the unit-cell volume panel show second-order Birch–Murnaghan fits.

The individual unit-cell parameters display distinctly different responses to pressure, demonstrating pronounced anisotropic compression behavior. In addition to the absolute changes shown in Figure 6, Figure S2 shows the relative changes with respect to the values at 0.1 GPa. Over the investigated pressure range, the *b* axis undergoes a fractional contraction of approximately 0.9% at all four temperatures, compared with approximately 0.4% for *a* and only about 0.3% for *c*. Thus, the *b* axis displays by far the strongest response to pressure. This observation agrees with the compression anisotropy previously established for MgCl_2_·10H_2_O-II. In that single-crystal diffraction study, analysis of the principal strains yielded linear compressibilities of *K*_1_ = 6.9(11) TPa^–1^, *K*_2_ = 14.5(18) TPa^–1^, and *K*_3_ = 24.6(14) TPa^–1^, corresponding to principal directions approximately along 0.5*a* + 0.9*c*, *a*, and *b*, respectively. The *b* axis was therefore also identified as the direction of maximum compressibility in the protonated material.

For the monoclinic MgCl_2_·10H_2_O-II, changes in *a* and *c* cannot generally be equated directly with principal strains because changes in the monoclinic angle β also contribute to deformation in the *ac* plane. The *b* axis, however, constitutes a principal strain direction by symmetry, making its identification as the most compressible direction particularly robust. The close correspondence between the pressure response of MgCl_2_·10D_2_O-II and the previously reported behavior of MgCl_2_·10H_2_O-II suggests that deuteration does not fundamentally alter the characteristic compression anisotropy of the decahydrate-II framework.

The previous structural analysis related this pronounced elastic anisotropy to the layered architecture and hydrogen-bonding network of MgCl_2_·10H_2_O-II. The *b* direction, which exhibits the greatest compressibility, lies within the structural layers, whereas the least compressible principal direction lies approximately along the layering direction (Figure 1b). Hydrogen-bonding interactions connect the Mg­(H_2_O)_6_ octahedra through additional water molecules along the *b* and *c* directions, including characteristic water-trimer configurations. The compression anisotropy observed here for the deuterated material therefore appears to reflect the same underlying structural characteristics as those identified previously for MgCl_2_·10H_2_O-II.

The pressure dependence of the unit-cell volumes at the four temperatures was analyzed using EosFit7,^36,37^ and the resulting fits are included in Figure 6 as solid lines. Each isotherm was fitted independently using a second-order Birch–Murnaghan equation of state.^40–42^ In the second-order Birch–Murnaghan formalism, the first pressure derivative of the bulk modulus, *K*_0_′, has the implied value of 4, leaving *V*_0_, the zero-pressure unit-cell volume, and *K*_0_, the isothermal bulk modulus at zero pressure, as the refined parameters. This treatment was considered appropriate for the relatively restricted pressure interval from 0.1 to 0.4 GPa, over which an independent refinement of *K*_0_′ would not be well constrained.

At 200 K, the fit yielded *V*_0_ = 1285.1(1.8) Å^3^ and *K*_0_ = 17.1(1.6) GPa. At 210 K, the corresponding values were *V*_0_ = 1286.2(1.9) Å^3^ and *K*_0_ = 17.2(1.7) GPa. At 220 K, *V*_0_ = 1287.9(2.3) Å^3^ and *K*_0_ = 16.4(1.9) GPa were obtained, while the 230 K isotherm yielded *V*_0_ = 1290.3(1.5) Å^3^ and *K*_0_ = 15.6(1.1) GPa. The four independently determined bulk moduli are mutually consistent within their uncertainties. Although the central values suggest a decrease from approximately 17.1 GPa at 200–210 K to 15.6 GPa at 230 K, the uncertainties overlap substantially. Consequently, the present data provide no statistically compelling evidence for a temperature dependence of *K*_0_ over the investigated 30 K temperature interval. This observation is important for the subsequent global *p*–*V*–*T* analysis because it indicates that the introduction of an additional pressure–temperature cross term to describe a temperature-dependent bulk modulus is not warranted by the available data.

The bulk moduli obtained from the individual isothermal fits can be compared with the value previously determined for the protiated analogue MgCl_2_·10H_2_O-II. A second-order Birch–Murnaghan fit to the protiated material at 220(2) K yielded *K*_0_ = 18.9(12) GPa.^20^ The present values of approximately 16–17 GPa for deuterated MgCl_2_·10D_2_O-II are somewhat smaller, nominally indicating a slightly greater compressibility of the deuterated material. However, this difference should be interpreted cautiously. In addition to the H/D isotopic substitution, the pressure ranges and experimental methods differ. The previous equation-of-state fit extended to pressures approaching 1 GPa and was based on single-crystal X-ray diffraction, whereas the present measurements span only 0.1–0.4 GPa and the unit-cell parameters were obtained from Le Bail refinements of the powder neutron diffraction data. The present comparison therefore does not provide sufficient evidence to assign the difference in *K*_0_ specifically to an isotope effect.

Nevertheless, the present results reinforce the previous conclusion that the decahydrate-II structure is relatively compressible. The *K*_0_ = 18.9(12) GPa value previously obtained for MgCl_2_·10H_2_O-II^20^ was comparable with those of other highly hydrated salt hydrates, including MgSO_4_·9D_2_O,^43^ MgSO_4_·11D_2_O,^44^ and Na_2_SO_4_·10D_2_O,^45^ with bulk moduli of 19.5(3), 19.9(4), and 19.6(1) GPa, respectively, and lower than that of the denser and less highly hydrated MgCl_2_·7H_2_O,^18^ for which *K*_0_ = 21.4(18) GPa was found. The values obtained here are therefore consistent with MgCl_2_·10D_2_O-II, likewise representing a relatively soft, highly hydrated framework.

The complementary temperature dependence of the unit-cell parameters and volumes at the four investigated pressures is shown in Figure 7. The unit-cell parameters and volumes all increase with increasing temperature at each pressure, indicating positive thermal expansion. The zero-pressure volumes obtained earlier from the four isothermal equation-of-state fits similarly increase systematically from 1285.1(1.8) Å^3^ at 200 K to 1290.3(1.5) Å^3^ at 230 K, also providing indication of the positive thermal expansion of MgCl_2_·10D_2_O-II.

**Figure 7:**
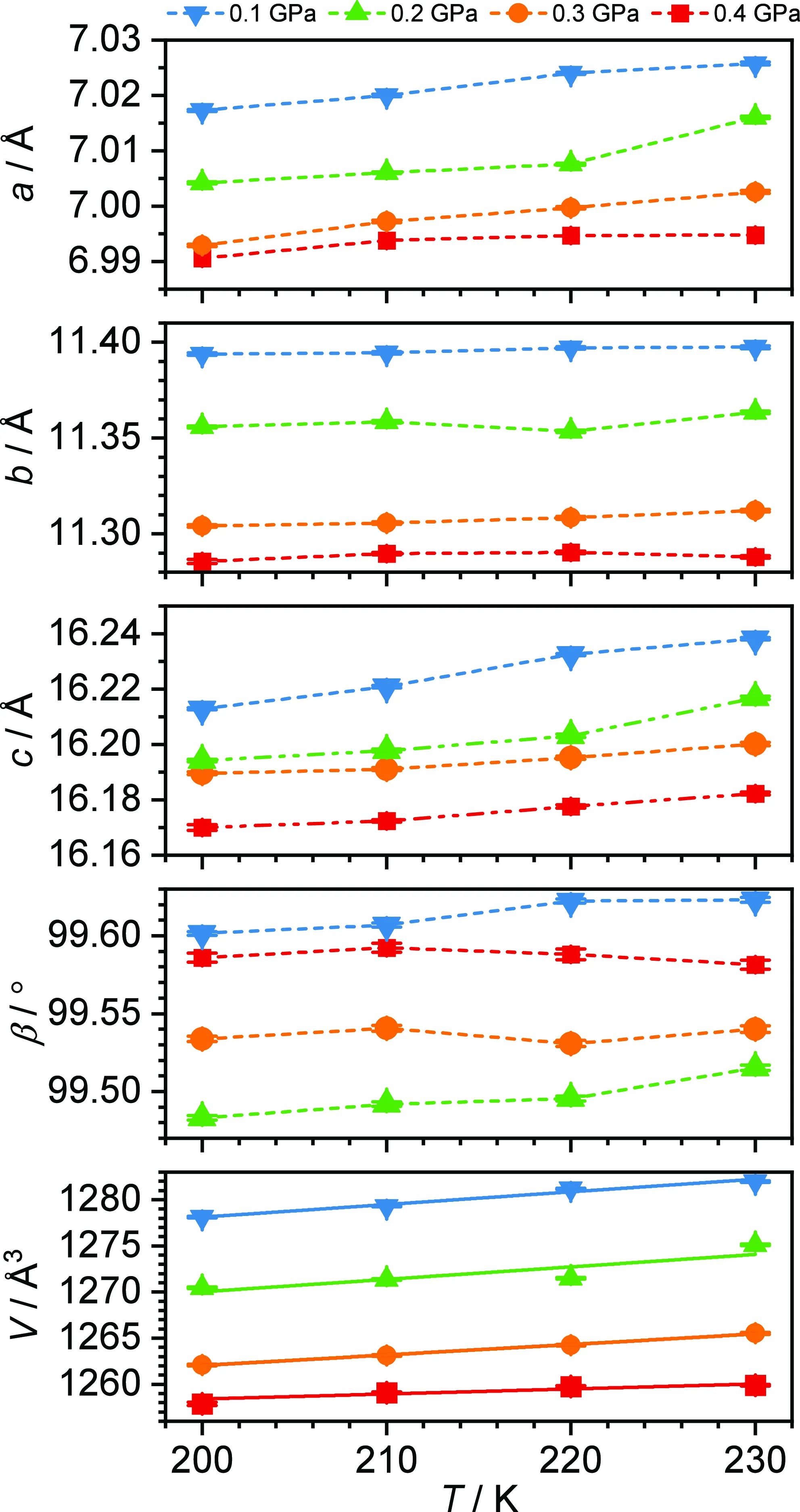
Unit-cell parameters and volumes of MgCl_2_·10D_2_O-II as a function of temperature at pressures of 0.1 GPa (blue downward-pointing triangles), 0.2 GPa (green upward-pointing triangles), 0.3 GPa (orange circles), and 0.4 GPa (red squares). Estimated standard deviations are indicated by error bars and are typically smaller than the symbol sizes. The dashed lines are guides to the eye, whereas the solid lines in the unit-cell volume panel show linear fits.

The temperature dependence of the individual unit-cell parameters is strongly anisotropic. To compare their thermal responses independently of the different absolute lengths of the crystallographic axes, the relative changes can be considered in terms of *a*/*a*_0_, *b*/*b*_0_, and *c*/*c*_0_, where the corresponding unit-cell parameters at 200 K at each pressure are taken as the reference values, as shown in Figure S3. Between 200 and 230 K, *b* increases by only approximately 0.02–0.07%, whereas the corresponding changes are approximately 0.06–0.17% for *a* and 0.07–0.16% for *c*. Thus, the *b* axis consistently displays the smallest fractional thermal expansion over the investigated pressure range.

Linear fits to the unit-cell parameters give approximate linear thermal-expansion coefficients of (1.9–5.3) × 10^–5^ K^–1^ along *a*, (0.7–2.4) × 10^–5^ K^–1^ along *b*, and (2.2–5.4) × 10^–5^ K^–1^ along *c*, depending on the pressure. Because these estimates are based on only four temperatures spanning a relatively narrow 30 K interval, their detailed pressure dependence should not be overinterpreted. In particular, the relative magnitudes of the expansion along *a* and *c* vary with pressure and cannot be assigned an unambiguous ordering over the complete pressure range. In contrast, the comparatively weak thermal response of *b* is consistently observed at all four pressures and therefore represents a robust feature of the data.

Interestingly, the anisotropy of the thermal expansion differs markedly from that of the compression anisotropy. The *b* direction, which exhibits the largest fractional contraction with increasing pressure, displays the smallest fractional expansion with increasing temperature. Thus, the thermal response cannot simply be regarded as the inverse of elastic compression. Instead, the different anisotropies presumably reflect the different ways in which pressure and temperature affect the hydrogen-bonded framework and molecular packing of the hydrate (Figure 1b). Pressure predominantly favors structural rearrangements that reduce the unit-cell volume, whereas thermal expansion can additionally be accommodated through changes in hydrogen-bond geometries, molecular orientations, and the relative arrangement of the hydrated ionic layers. A more detailed microscopic interpretation of the thermal anisotropy would, however, require structural Rietveld refinements as a function of temperature rather than unit-cell parameters obtained from Le Bail fits alone. Because of preferred orientation effects in our sample, this is unfortunately not possible.

Following analysis of the four individual isotherms, all *p*–*V*–*T* data points were fitted simultaneously to obtain a global thermal equation of state. A second-order Birch–Murnaghan compression model was combined with a Berman thermal-expansion model^46^ using a reference temperature of 220 K. The zero-pressure volume *V*_0_, bulk modulus *K*_0_, and first thermal-expansion coefficient α_0_ were refined. *K*_0_′ was again fixed at its implied second-order Birch–Murnaghan value of 4, while the higher-order thermal-expansion coefficient α_1_ was fixed at zero. No *p*–*T* cross term was included. The latter choice is supported by the individual earlier isothermal fits, which show no statistically significant variation of *K*_0_ between 200 and 230 K.

The global *p*–*V*–*T* refinement yielded *V*_0_(220 K) = 1288.4(7) Å^3^, *K*_0_ = 16.1(6) GPa, and α_0_ = 9.95 (1.97) × 10^–5^ K^–1^. The global value of *K*_0_ lies within the uncertainty ranges of all four independently determined isothermal values and is therefore fully consistent with the individual compression analyses. Similarly, the positive thermal-expansion coefficient obtained from the global refinement agrees with the systematic increase in the independently determined *V*_0_ values with increasing temperature. The agreement between the two approaches indicates that the relatively simple second-order Birch–Murnaghan–Berman model captures the principal compression and thermal-expansion behavior of MgCl_2_·10D_2_O-II over the investigated *p*–*T* range.

The absence of a statistically significant variation in the individual *K*_0_ values also supports the decision not to include a *p*–*T* cross term in the global fit. Likewise, the relatively narrow temperature interval does not justify refinement of the higher-order Berman coefficient α_1_. Retaining these parameters as fixed therefore avoids overparameterization of the 16-point *p*–*V*–*T* data set. It should also be noted that, given the somewhat limited pressure range and number of data points available for each isotherm, some correlation between the fitted *V*_0_ and *K*_0_ values cannot be excluded.

The uncertainties of the unit-cell parameters and volumes used in the equation-of-state analysis originate predominantly from the Le Bail refinements and consequently represent primarily the statistical precision with which the unit-cell dimensions can be determined from the diffraction patterns. They do not necessarily encompass all sources of systematic experimental uncertainties. The uncertainties of the equation-of-state parameters were therefore rescaled by EosFit according to the observed scatter of the measurements about the global equation of state. This treatment provides a more realistic representation of the precision with which the equation-of-state parameters can be determined from the present data set than would be obtained from the formal Le Bail estimated standard deviations alone.

Overall, the present *p*–*T* measurements complement the previous high-pressure investigation of MgCl_2_·10H_2_O-II in two important respects. First, they demonstrate that the characteristic anisotropic compression of the decahydrate-II framework is retained in MgCl_2_·10D_2_O-II, with the *b* axis contracting by approximately 0.9% between 0.1 and 0.4 GPa, compared with approximately 0.4% for *a* and 0.3% for *c*. The *b* axis therefore again represents the most compressible crystallographic direction. Second, the measurements reveal a pronounced and distinctly different anisotropy in the thermal expansion. Most notably, the *b* direction is simultaneously the most compressible direction under pressure and the direction exhibiting the smallest fractional thermal expansion. The responses of the structure to pressure and temperature are therefore strongly anisotropic but are not simply inversely related. Together with the relatively low bulk modulus of *K*_0_ = 16.1(6) GPa and the volumetric thermal-expansion coefficient of α_0_ = 9.95 (1.97) × 10^–5^ K^–1^, these observations further illustrate the important role of hydrogen-bonding interactions and molecular packing in controlling the thermoelastic behavior of this highly hydrated salt framework.

## Conclusions

In this work, we investigated the crystallization of a nearly saturated MgCl_2_/D_2_O solution with a molar ratio of 1:10.3 under isobaric conditions from ambient pressure to 0.4 GPa using *in situ* neutron diffraction. At ambient pressure, substantial supercooling was observed, with crystallization occurring only between 240 and 230 K. The principal crystalline product upon cooling was MgCl_2_·12D_2_O, while the difference between its hydration state and the bulk sample composition suggests that a salt-rich fraction remained as a metastable glassy solution. Upon heating, MgCl_2_·8D_2_O formed initially from this residual fraction and subsequently through the peritectic decomposition of the dodecahydrate. These observations are consistent with the known ambient-pressure MgCl_2_–H_2_O phase diagram but highlight the important role of supercooling, kinetic effects, and metastability in determining the phases obtained during crystallization of concentrated MgCl_2_ solutions.

A relatively modest increase in pressure to 0.1 GPa fundamentally changes the crystallization pathways. Throughout the investigated pressure range from 0.1 to 0.4 GPa, MgCl_2_·10D_2_O-II is the dominant MgCl_2_ hydrate formed upon isobaric cooling. This demonstrates that the recently discovered decahydrate-II polymorph^20^ can crystallize directly from the liquid at considerably lower pressures and along much simpler *p*–*T* pathways than those employed in its original discovery. The isobaric crystallization pathway is expected to be highly reliable, which opens the way for preparing large amounts of pure MgCl_2_·10D_2_O-II for further spectroscopic characterizations that are relevant in the context of the remote detection of this hydrate.

At 0.3 and 0.4 GPa, an additional weak diffraction feature appears upon further cooling and is consistent with the formation of either ice II or ice III from a small water-rich residual fraction. This observation would be consistent with the existence of a low-pressure eutectic between MgCl_2_·10D_2_O-II and one of these high-pressure ice phases.

The pressure- and temperature-dependent diffraction measurements additionally provide a thermoelastic characterization of MgCl_2_·10D_2_O-II over the investigated 0.1–0.4 GPa and 200–230 K ranges. The structure displays pronounced anisotropic compression, with the *b* axis being substantially more compressible than the *a* and *c* axes, consistent with the behavior previously reported for MgCl_2_·10H_2_O-II.^20^ In contrast, the *b* axis exhibits the smallest fractional thermal expansion, demonstrating that the responses of the structure to pressure and temperature are strongly anisotropic but are not simply inversely related. A global *p*–*V*–*T* analysis yields a zero-pressure bulk modulus of *K*_0_ = 16.1(6) GPa and a volumetric thermal-expansion coefficient of α_0_ = 9.95 (1.97) × 10^–5^ K^–1^ at a reference temperature of 220 K. The relatively low bulk modulus is consistent with the highly hydrated and comparatively soft nature of the decahydrate-II framework and with the behavior of other highly hydrated salt hydrates.^43–45^

These findings have important implications for the mineralogy of icy moons. Pressures between 0.1 and 0.4 GPa are readily encountered within the hydrospheres of large icy worlds,^1^ and, notably, MgCl_2_·10H_2_O-II crystallizes in our experiments already at 0.1 GPa, well within the pressure range expected even for Europa’s subsurface ocean, where pressures may only reach approximately 0.2 GPa at the seafloor.^47,48^ For comparison, the maximal pressure at the seafloor of Ganymede has been estimated at around 1.6 GPa.^1^ The direct crystallization of MgCl_2_·10H_2_O-II under these conditions therefore suggests that this phase may be considerably more relevant to Europa and other icy-moon hydrospheres than previously recognized. More generally, the striking change from MgCl_2_·12H_2_O at ambient pressure to MgCl_2_·10H_2_O-II between zero pressure and only 0.1 GPa demonstrates that even relatively modest pressures can fundamentally alter the salt-hydrate assemblages produced by freezing MgCl_2_-bearing brines. Ambient-pressure phase diagrams alone may therefore provide an incomplete picture of the mineralogy expected within icy-moon hydrospheres. Together with the recent discovery of previously unknown stable and metastable hydrates in the NaCl–H_2_O system,^3–5^ our results highlight the need to investigate the crystallization of salty aqueous solutions directly under planetary-relevant pressure and temperature conditions. Extending such studies to more complex, multicomponent brines representative of predicted subsurface ocean compositions will be an important next step toward linking salt-hydrate mineralogy to the composition, thermal history, and transport processes of icy-moon hydrospheres.

## Supplementary Material



## Notes

Time-of-flight diffraction data of the empty TiZr can (Figure S1), relative changes in the lattice parameters and unit-cell volumes of MgCl_2_·10D_2_O-II as a function of pressure (Figure S2) and temperature (Figure S3), and neutron diffraction data upon isobaric heating at 0.2 GPa (Figure S4) (PDF)
