# Supplementary material for: Isobaric Crystallization of Aqueous Magnesium Chloride Solutions at Icy Moon Conditions
Source: J Phys Chem A. 2026 Oct 22;130(44):8899–910. doi: 10.1021/acs.jpca.6c05426 (PMC13746819; doi:10.1021/acs.jpca.6c05426)
Supplement: Supplementary file 1 [file jp6c05426_si_001.pdf]

Supporting Information

Isobaric Crystallization of Aqueous Magnesium Chloride Solutions at Icy Moon Conditions

Milz L. Beaumont,[1] Jinjie Li,[1] Miroslava Novoveska,[1] Ines E. Collings,[2] Craig L. Bull,[3] Christoph G. Salzmann[1*]

[1] Department of Chemistry, University College London, 20 Gordon Street, London WC1H 0AJ, United Kingdom.

[2] Natural History Museum, Cromwell Road, South Kensington, London SW7 5BD, United Kingdom.

[3] ISIS Pulsed Neutron and Muon Source, STFC Rutherford Appleton Laboratory, Harwell Campus, Harwell OX11 0QX, United Kingdom.

Corresponding Author

* c.salzmann@ucl.ac.uk

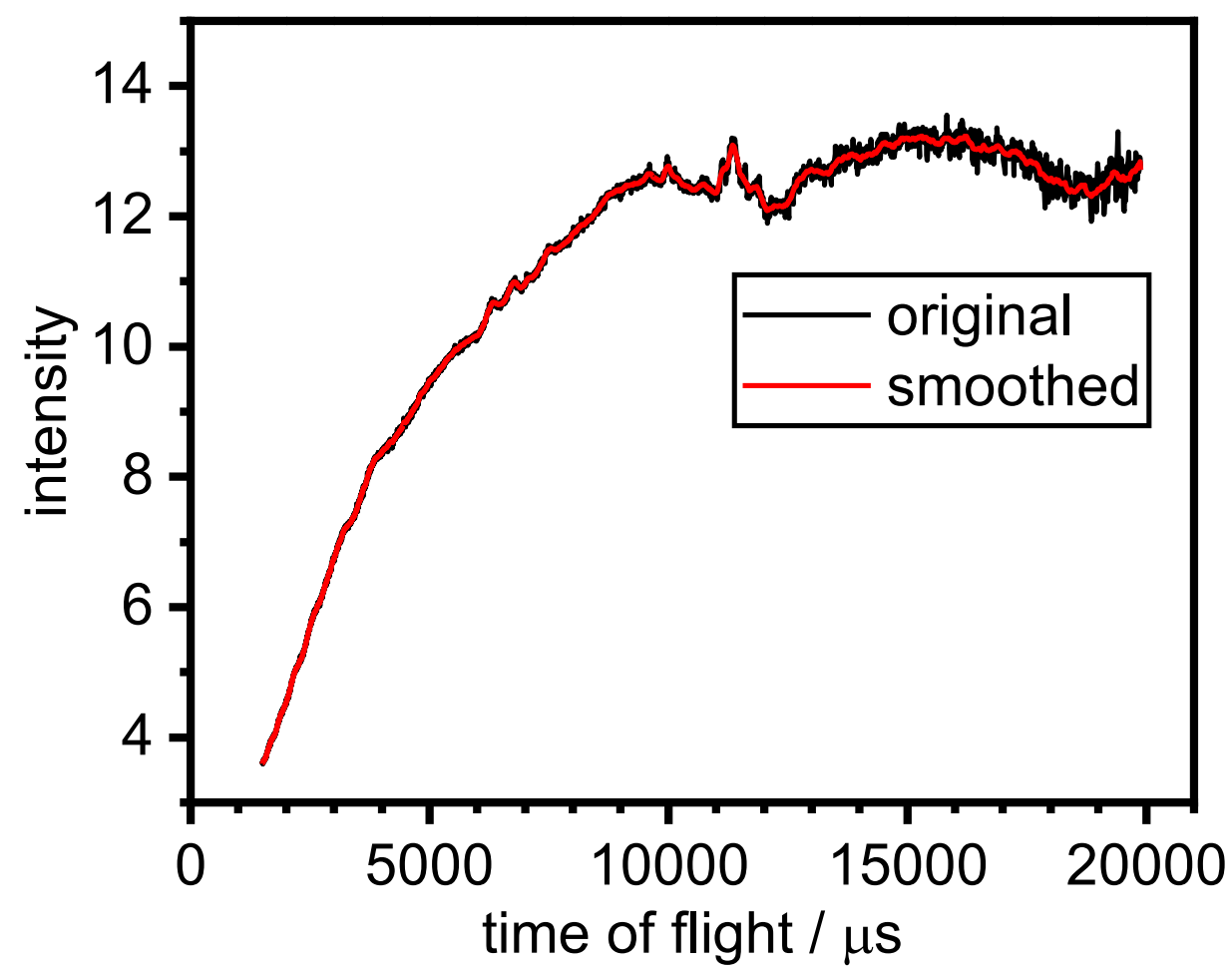

Figure S1. Time-of-flight diffraction data of the empty TiZr can. The as-recorded data are shown as a black line and the smoothed data (adjacent averaging with 20-point window) as a red line.

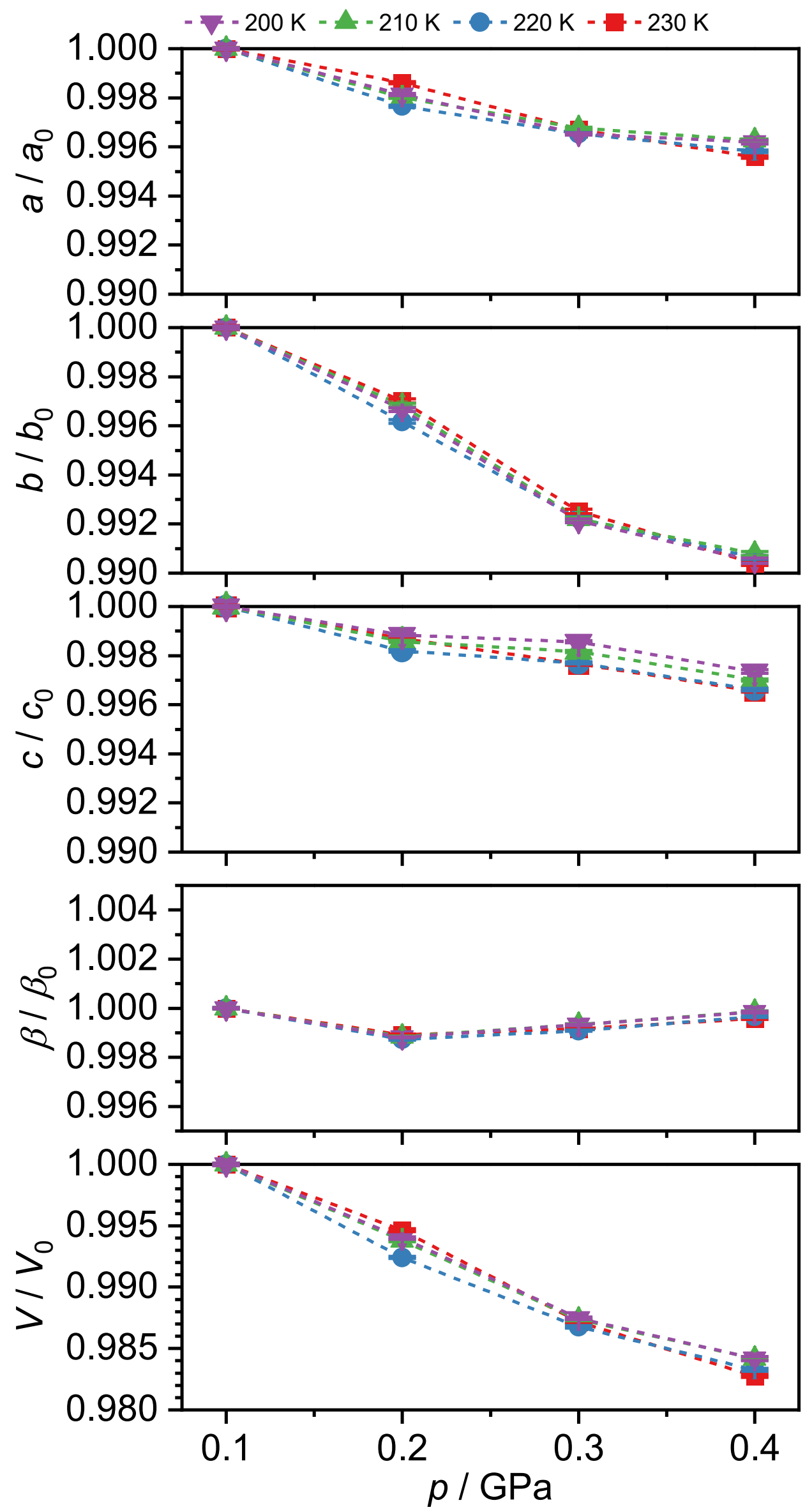

Figure S2. Relative changes in the unit-cell parameters and volumes of $MgCl_2 \cdot 10D_2O$-II with respect to their values at 0.1 GPa and temperatures of 200 K (purple downward-pointing triangles), 210 K (green upward-pointing triangles), 220 K (blue circles) and 230 K (red squares). Estimated standard deviations are indicated by error bars and are typically smaller than the symbol sizes.

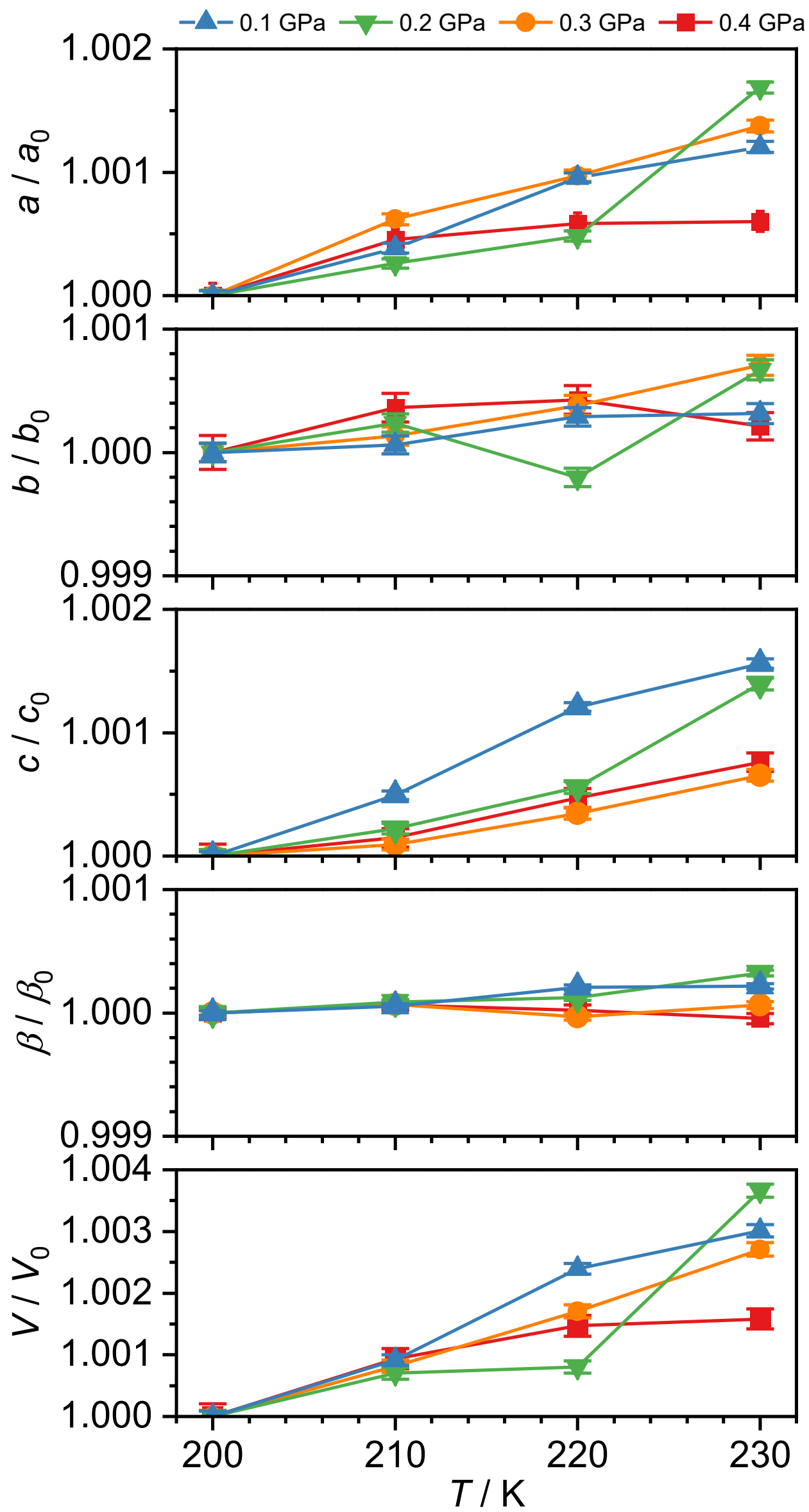

Figure S3. Relative changes in the unit-cell parameters and volumes of MgCl2·10D2O-II with respect to their values at 200 K and pressures of 0.1 GPa (blue upward-pointing triangles), 0.2 GPa (green downward-pointing triangles), 0.3 GPa (orange circles) and 0.4 GPa (red squares). Estimated standard deviations are indicated by error bars and are typically smaller than the symbol sizes.

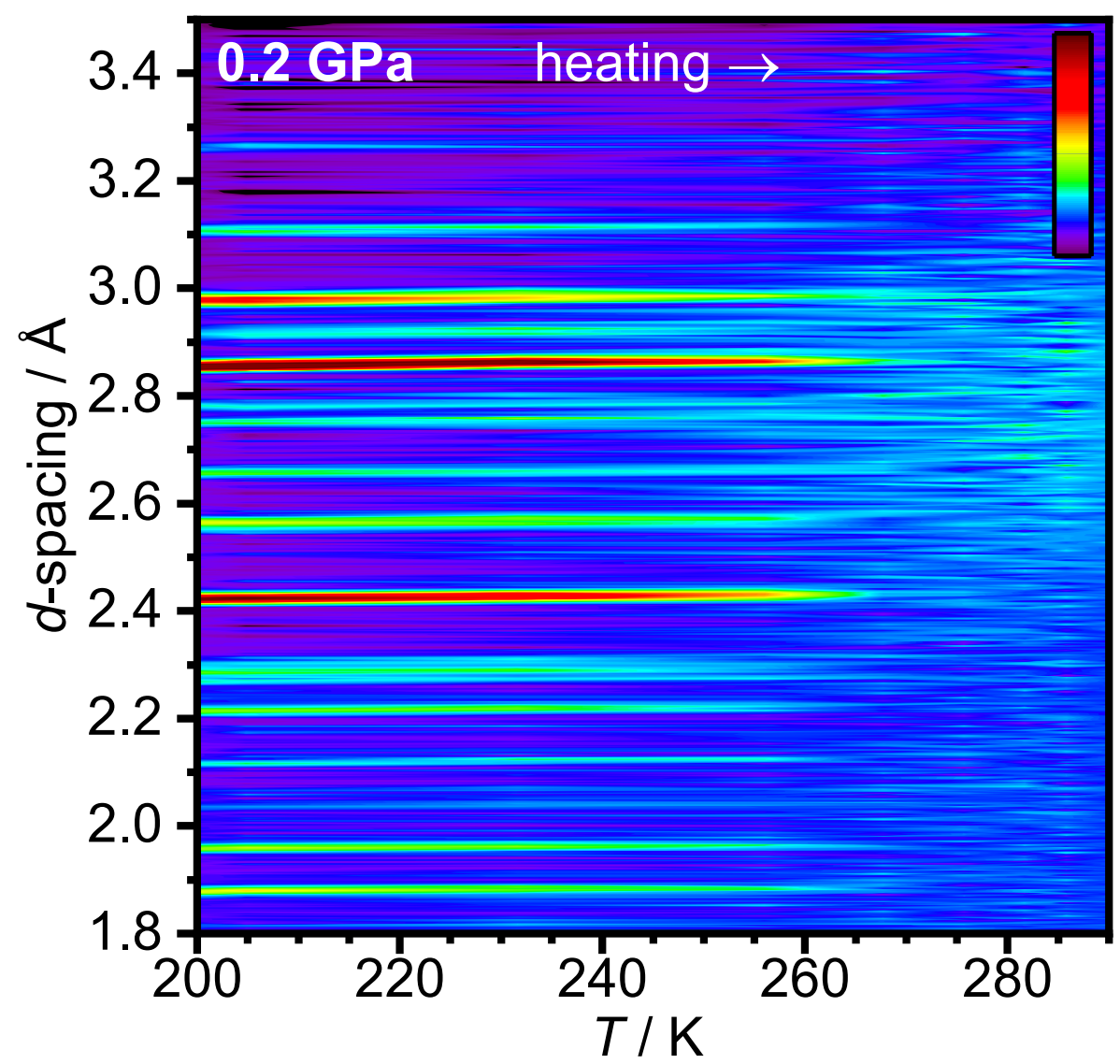

Figure S4. Neutron diffraction data collected upon isobaric heating of a 1:10.3 $MgCl_2$:D_2O sample at 0.2 GPa from 200 to 290 K.
